# Neuroanatomical correlates of visual car expertise

**DOI:** 10.1016/j.neuroimage.2012.05.017

**Published:** 2012-08-01

**Authors:** Sharon Gilaie-Dotan, Assaf Harel, Shlomo Bentin, Ryota Kanai, Geraint Rees

**Affiliations:** a Institute of Cognitive Neuroscience, University College London, London, UK; b Wellcome Trust Centre for Neuroimaging, University College London, London, UK; c Department of Psychology, Hebrew University of Jerusalem, Jerusalem, Israel; d Laboratory of Brain and Cognition, National Institute of Mental Health, National Institutes of Health, Bethesda, MD, USA; e Interdisciplinary Center for Neural Computation, Hebrew University of Jerusalem, Jerusalem, Israel

**Keywords:** Visual expertise, VBM, PFC

## Abstract

Expertise in non-visual domains such as musical performance is associated with differences in gray matter volume of particular regions of the human brain. Whether this is also the case for expertise in visual object recognition is unknown. Here we tested whether individual variability in the ability to recognize car models, from novice performance to high level of expertise, is associated with specific structural changes in gray matter volume. We found that inter-individual variability in expertise with cars was significantly and selectively correlated with gray matter volume in prefrontal cortex. Inter-individual differences in the recognition of airplanes, that none of the participants had expertise with, were correlated with structural variability of regions bordering the visual cortex. These results highlight the role of prefrontal regions outside the visual cortex in accessing and processing visual knowledge about objects from the domain of expertise and suggest that expertise in visual object recognition may entail structural changes in regions associated with semantic knowledge.

## Highlights

► We examined neuroanatomical correlates of expertise in car recognition. ► The neural structure of prefrontal regions predicted expertise in car recognition. ► No structural correlates within ventral occipital cortex were found for car expertise.

## Introduction

Humans are generally very good at visually recognizing objects in the environment. Yet, this visual ability can be improved even more for objects in a particular domain by learning and accumulating experience. Birdwatchers, car buffs, and dog show referees are all examples of people who acquired the expertise needed to distinguish between highly similar exemplars of very specific object categories. The neural basis of such intentionally acquired visual expertise remains unclear. One prominent view suggests that acquired expertise in making fine within-category distinctions is based on developing global perceptual strategies, similar to the characteristics of naturally-developed expertise for face individuation and recognition (Gauthier and Tarr, 2002; Gauthier et al., 2003; Richler et al., 2011; Wong and Gauthier, 2010a), for a review see Bukach et al. (2006). Functional magnetic resonance imaging (fMRI) studies examining the neural basis of expertise in visual object recognition from this perspective have focused on the ventral visual cortex with particular interest in the fusiform gyrus, whose activity is associated with global processing (Bilalic et al., 2011b; Gauthier et al., 1999, 2000b; Gilaie-Dotan et al., 2010; van der Linden et al., 2008; Wong et al., 2009). Yet, other areas across occipito-temporal cortex have also been implicated in visual object recognition (Bilalic et al., 2011a; Harley et al., 2009; Op de Beeck et al., 2006).

However, there are two lines of neuropsychological findings suggesting that in addition to ventral visual cortex, other brain regions may also support expert object recognition. First, patients can retain expert car recognition despite lesions to ventromedial occipito-temporal cortex (Sergent and Signoret, 1992) and are even able to learn new visual expertise (McNeil and Warrington, 1993). Second, damage to regions in prefrontal and anterior temporal cortex is also associated with deficits in visual object recognition (Damasio et al., 2004) suggesting that regions other than the occipito-temporal cortex may also be involved in object recognition and, by extension, associated with expertise with visual recognition of specific object categories (Jefferies et al., 2011).

Along these lines, recent investigations of expertise in object recognition propose that visual expertise manifests as widespread activity distributed across many cortical regions rather than confined to specific category-selective regions of ventral visual cortex (Bilalic et al., 2010; Harel et al., 2010; Wong and Gauthier, 2010b). For example, we have previously shown that when car experts are engaged in the recognition of cars, the network of cortical regions that distinguishes them from non-expert participants extends beyond visual cortex, reaching into frontal and parietal cortex (Harel et al., 2010). Other studies show that experts use a broader spatial detection window for their preferred category, their short term visual memory capacity is enhanced for that category, and their categorization might be influenced by top-down signals (Curby et al., 2009; Harel et al., 2011; Hershler and Hochstein, 2009). These findings suggest that, in addition to perception, expert processing is evident in cognitive processing that involves attention, working memory, and the application of semantic knowledge; together, these processes determine the distribution of expertise-related brain activity (Bilalic et al., 2011a), for a similar view, see (Pylyshyn, 1999).

A complementary approach to study the neural manifestations of expertise is to measure how brain structure differs with expertise as opposed to measuring differences in brain activation. Expertise-related structural changes in the brain allow assessment of consequences of long-term expertise on gray matter structure across the entire brain, independently of the context set by a particular experimental task. One successful and widely used approach to assess possible relationships between brain structure and behavior is voxel-based morphometry (VBM, Ashburner and Friston, 2000). VBM associates inter-individual differences in gray matter density with inter-individual variation along a behavioral or perceptual measure (Kanai and Rees, 2011). Long-term real-world expertise in several non-visual domains, such as spatial navigation, musical performance and fluency in a second language is associated with marked changes in brain structures of specific regions (Gaser and Schlaug, 2003; Maguire et al., 2000; May, 2011; Woollett and Maguire, 2011). Whether structural changes are associated with long-term expertise in visual object recognition has not yet been studied. To this end, we hypothesized that, as with other non-visual domains, individual variability in visual experience with recognizing an object category (culminating in expertise) might be associated with neural structural changes in the human brain (Kanai and Rees, 2011).

**Fig. 1:**
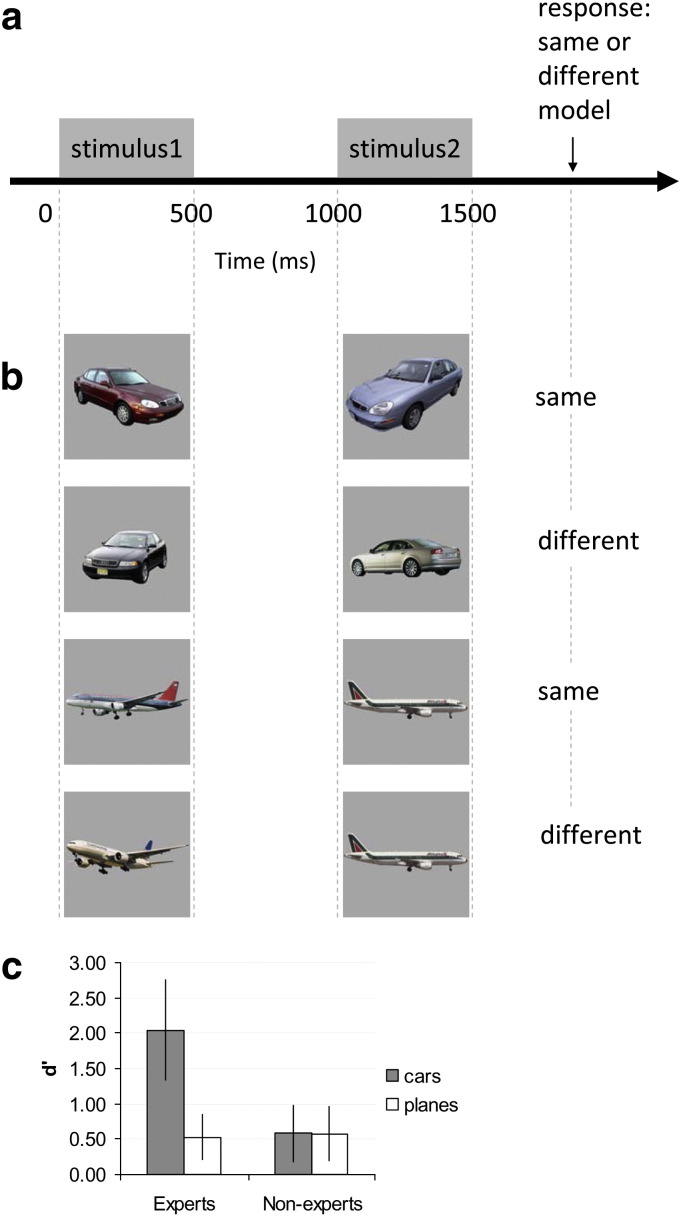
Behavioral paradigm. (a) Trial time line to determine car expertise. (b) Examples of typical stimuli used in the car model discrimination (top two rows) or airplane discrimination (bottom two rows) tasks, with the expected correct response in each example. In the car model discrimination task, participants had to determine whether two presented car images were of the same car model regardless of color, viewpoint and production year. Likewise in the airplane discrimination task they had to decide if the planes are of the same plane manufacturer. See further details in Methods. (c) Behavioral performance of car experts (participants that performed at 83% accuracy or higher on the car discrimination experiment) and non-experts (novices) for cars and planes. Note that this analysis distinguishing experts and novices is provided only to convey that the car experts outstood in their car recognition abilities, but not in the control plane task. Importantly, all other analyses in this study including the structural correlation analysis treated car expertise as a *continuous* variable and did not compare between novices and experts. Discrimination sensitivities (d′) for cars (gray) and planes (white) by car experts (n = 12, left) and novices (n = 9, right). Car experts’ performance for cars was significantly higher than for planes (1-tailed paired *t*-test, p = 0.000019, t(11) = 7.134, n = 12) and significantly higher than novices’ performance for cars (1-tailed unequal sample sizes and unequal variance *t*-test, p < 0.001, t (18) = 5.981, n_1_ = 12, n_2_ = 9). Novices’ performance was similar for both cars and planes (2-tailed paired *t*-test, p = 0.993, t(8) = 0.0087, n = 9), and there was no difference between car experts and novices in the performance for planes (2-tailed unequal sample sizes and equal variance *t*-test, p = 0.7519, t(19) = 0.32, n_1_ = 12, n_2_ = 9). Error bars, S.D.

To explore this hypothesis, we pooled together a group of individuals who were experts in recognizing the models of cars presented to them visually along with a group of other individuals who showed no particular expertise in this task. Car expertise was chosen because car experts are relatively frequent, because this type of expertise has been amply studied (e.g. Bukach et al., 2010; Gauthier et al., 2003; Harel et al., 2011; Hershler and Hochstein, 2009; Rossion and Curran, 2010) and shows sufficient inter-individual variability to allow us to test our hypothesis. Participants were recruited based on their self-reported interest in cars, and were then formally assessed for their car model recognition ability and semantic knowledge of recent car models. After selecting the experts (see below), we recruited a group of control participants, matched in age and education with the car experts, and also assessed their car model recognition ability using the same test (for a similar approach see Gauthier et al., 2000a, 2000b; Xu, 2005). Thus, the performance of the participants in the car model recognition task (see Methods and Fig. 1) formed a continuum of expertise from novice to expert that allowed us to examine whether variability in car expertise (as measured in this study) correlated with variability in neuroanatomical structure in the brain, as revealed by structural MRI. Moreover, if such correlation was found it might also delineate the neuroanatomical structures that might be associated with real world, gradually acquired visual expertise for cars and determine whether such regions were located within the visual category-selective cortex. Importantly, in order to determine whether the association between brain structure and visual recognition performance was specific to the category of expertise (cars), we also performed such an assessment for a control task, in which the participants discriminated among exemplars of a similarly complex man-made category of objects (airplanes) for which none of the participants was particularly an expert (see Methods).

## Methods

### Participants

The participants were 12 men who defined themselves as car experts (aged 24.3 ± 5.18 (s.d.) years) and 9 men, self-defined car-naives (aged 25.9 ± 2.37 (s.d.)) who were matched in education with the experts. All of the participants participated in a previous study ((Harel et al., 2010), see Supplementary Material for details). The car experts were recruited among volunteers who responded to messages posted in car forums on the Internet. To formally assess the car expertise of all candidates we used a same-different car model recognition task (see details below) inspired by (Gauthier et al., 2000a, 2000b; Xu, 2005), and an additional semantic task which was aimed at assessing the extent of their knowledge and familiarity with the cars presented in the model recognition task (not reported here). Expertise in the current study was determined by scoring an accuracy level of 83% or above in the car recognition task or above 71% in a more difficult recognition task (using less familiar car models). Furthermore, all self-defined experts displayed extensive knowledge about car models in the semantic task. Importantly, all “experts” reported a life-long interest in cars and displayed extensive knowledge about the cars presented in this study.

All the participants completed the behavioral experiments (same-different recognition tests with cars and airplanes—see below), and structural MRI scans. They were all healthy and had normal or corrected-to-normal vision. Written informed consent to participate in the experiment was obtained from all the participants according to the Tel Aviv Sourasky Medical Center ethics committee that approved the experimental protocol.

### Expertise testing procedures

The same-different car model recognition test used to determine the level of car expertise for each participant was fully described in a previous study (Harel et al., 2010). On each trial, participants determined whether two images of cars presented sequentially (for 500 ms each with 500 ms ISI) were of the same model (e.g. Honda Civic) or not. The two images on each trial were always of the same make of car (e.g. Honda), but were physically different, as they differed in year of production, color, angle and direction of presentation (see Fig. 1 for representative stimuli in each condition). Thus, while for ‘different model’ trials the two images were of the same car-maker but different models (e.g. VW Golf and VW Passat), for ‘same model’ trials the two images depicted two cars of the same model but differed physically in many aspects (e.g. viewpoint, car color, see Fig. 1b). The experiment consisted of 80 trials (40 ‘same model’ trials, 40 ‘different model’ trials), which were based on 160 different car images. No identical pairs or images were repeated throughout the experiment. The car images were of frequently encountered models from recent years. Note that the participants’ score on this task was later used as a predictor in the structural brain analysis for car expertise (VBM, see below).

To test whether inter-individual variability in car expertise was category-specific, participants also performed an analogous experiment in which they were instructed to match images of passenger airplanes. The passenger planes experiment was prepared and displayed in the same manner as the car experiment (e.g. based on 160 different airplane images, see Fig. 1b). The order of the trials within each experiment varied across participants.

### Stimuli

The stimuli were cars or airplanes centered on a uniform gray background (see Fig. 1). They were 360 × 360 pixels in size corresponding to a visual angle of approximately 9° × 9°, and were presented in a darkened room using the E-prime software (Psychology Software Tools, Inc., Sharpsburg, PA) on a 17‐inch CRT monitor with a 60 Hz refresh rate. Responses were provided using two different keys on the keyboard.

### MRI data acquisition

High resolution MR anatomical images providing whole‐brain coverage were acquired for each of the participants on a 3T GE scanner in the Tel Aviv Sourasky Medical Center. A spoiled gradient (SPGR) sequence was acquired (TR = 5.576 ms, TE = 1.388 ms, flip angle 12°, FOV 250 × 250 mm^2^, matrix size 256 × 256, slice thickness 1.0 mm, voxel size 1.00 × 0.98 × 0.98 mm^3^, 154–162 slices).

### Structural MRI voxel-based morphometry analyses

**Fig. 2:**
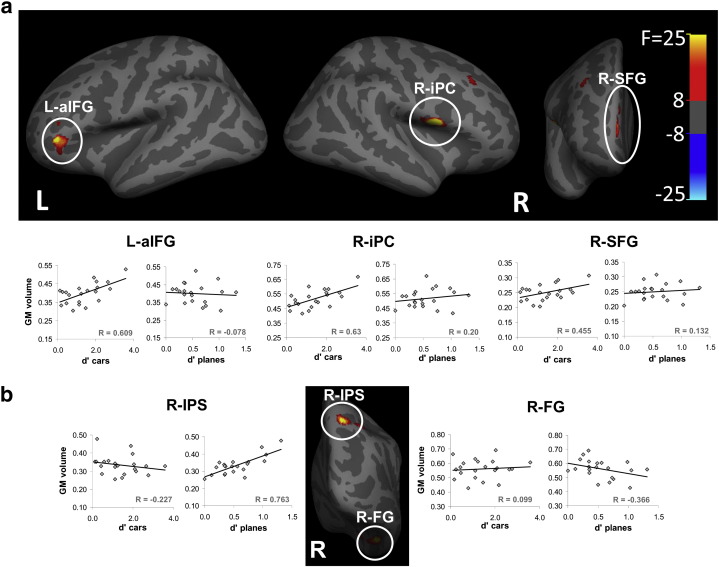
Neuroanatomical changes associated with visual car expertise. Red to yellow patches represent brain regions where neural structure significantly correlated with visual expertise in cars, or with a control category of planes, presented on inflated brains. (a) Frontal regions with neural structure associated with visual expertise including right inferior precentral (R-iPC), left anterior inferior frontal gyrus (L-aIFG) and right superior frontal gyrus (R-SFG), on lateral and frontal views. To show that the correlations are not driven by outliers we provide accompanying scatter plots between neural volume and individual performance (cars on left, planes on right, see Table 1 and Methods) that are for illustration only and should not be used for inference (circular reasoning, as these regions were identified as statistically significant in the whole‐brain analysis depicted above and described in the Methods). (b) Regions with neural structure associated with performance on the control task (airplanes) including right intraparietal sulcus (R-IPS) and right fusiform gyrus (R-FG), following conventions of (a). The color scale (right) indicates the F statistics of the structural correlates according to the VBM analysis (see also Table 1).

For each participant the structural MR images were first segmented to gray matter (GM) and white matter (WM) using the segmentation tools in SPM8 (http://www.fil.ion.ucl.ac.uk/spm). Subsequently, we performed Diffeomorphic Anatomical Registration through Exponentiated Lie Algebra (DARTEL) in SPM8 for inter-subject registration of the GM images (Ashburner, 2007; Ashburner and Friston, 2000). In this co-registration preprocessing, local GM volumes were conserved by modulating the image intensity of each voxel by the Jacobian determinants of the deformation fields computed by DARTEL. The registered images were then smoothed with a Gaussian kernel (FWHM = 8 mm) and transformed to the MNI stereotactic space using affine and non-linear spatial normalization implemented in SPM8 for multiple regression analysis. Multiple regression analyses were performed using SPM5. The covariates of interest included in the model were the level of car expertise as assessed by the sensitivity of the participants in the car discrimination (d′) and the discrimination sensitivity for the control category of planes (d′). The age of the participants and global gray matter volume (following ANCOVA normalization) were included in the design matrix as covariates of no interest, and were thus regressed out. F contrasts including performance-level in the car expertise (cars d′, see Fig. 2a) or the performance-level in the airplane expertise task (d′, see Fig. 2b) or both (cars d′ and airplanes d′, see Supp. Table 1) were applied first with p < 0.001 uncorrected as the criterion to detect voxels with significant correlation to individual's performance-level. Importantly, since both performance measures (d′ cars and d′ airplanes) were included in the design matrix, for each F contrast, the predictors not included in that contrast were treated as effects of no interest and regressed out in the analysis. For example, when only car expertise level was included (Fig. 2a), performance on the control task was regressed out. Since structural images display local variation in smoothness, standard applications of cluster-based random field theory are inappropriate (Hayasaka et al., 2004). Therefore non-stationary whole-brain cluster-level correction was applied using the ‘Non-Stationary Cluster Extent Correction for SPM’ toolbox (http://fmri.wfubmc.edu/cms/NS-General (Hayasaka et al., 2004)). We report (Fig. 2, Table 1, Supp. Table 1 and Supp. Fig. 1) only the results of clusters that survived this non-stationary correction for multiple comparisons across the whole brain at a threshold of p (corrected) < 0.05. Specific statistical details are provided in Table 1. No cluster size cutoff was applied to any of the analyses. The only analysis that we report at an uncorrected level (p < 0.001) follows applying a more lenient threshold aimed at examining neural structure correlates of visual car expertise in ventral visual cortex. In this case the F contrast including the expertise level was applied as above, and we examined the uncorrected p values at a threshold of p < 0.001.

To verify that the observed correlations were not driven by outliers in the data, peak gray matter densities were extracted from each significant cluster using the MarsBar toolbox (http://marsbar.sourceforge.net , M. Brett, J. Anton, R. Valabregue, and J. Poline. Human Brain Mapping conference, Japan, 2002) and plotted against individual performance. Note that while they are plotted in Fig. 2 and Supp. Fig. 1, these plots are not to be used for statistical inference to avoid circular reasoning (Kriegeskorte et al., 2009).

### Structural MRI threshold-free cluster enhancement (TFCE) analyses

Since the statistical method described above depends on the cluster-defining *t* and the assumption of normal distribution of data, we further performed the same regression analysis using a non-parametric permutation test with the Threshold-Free Cluster Enhancement (TFCE, (Smith and Nichols, 2009)) method, as implemented in the FSL package. Briefly, in this permutation test, we computed the significance of the correlations based on 5000 surrogate samples generated by permutation of the original data. This allowed us to make inferences on statistical maps without any assumptions about the null distribution of the data. Importantly, the TFCE approach allowed us to compute cluster-level statistics without arbitrarily defining the threshold for clusters. This additional analysis was performed to demonstrate the robustness of the results that did not depend on specific parameter settings or assumptions underlying the statistical tests.

## Results

As expected, car expertise varied widely across the participants from ‘novices’ to ‘experts’ (0.12 ≤ d′_cars_ ≤ 3.58, mean = 1.41 ± 0.94 s.d., n = 21, see Fig. 1c), while performance on the control task with airplanes was poorer and varied less across participants (0 ≤ d′_planes_ ≤ 1.31, mean = 0.55 ± 0.38 s.d., n = 21, see also Fig. 1c). There was no significant correlation between the performances on cars and airplanes (r = − 0.141, t(19) = − 0.622, p > 0.50).

Voxel-based morphometry (VBM) analysis was used to explore the correlation between the local gray matter volume across the whole brain and car expertise (see Methods). Regions showing significant correlations between neural structure and car expertise at a conventional statistical threshold (p < 0.05 corrected for multiple comparisons across the whole brain) were found in frontal regions including the right inferior precentral sulcus (bordering the upper insula), left anterior inferior frontal gyrus, and right superior frontal gyrus (see Fig. 2a, and Table 1 for a full list and details of loci). No significant regions were found in the occipito-temporal visual cortex.

To further examine whether the absence of correlations between the local gray matter volume and car expertise in visual regions was due to our stringent statistical threshold, we also examined the data at a more lenient threshold (p < 0.001 uncorrected for multiple comparisons) to detect sub-threshold correlations. This examination, however, did not reveal any other regions in which brain structure correlated with visual car expertise. Additional region-of-interest analysis to examine specifically whether the gray matter structure of functionally defined face-selective FFA was associated with car expertise did not reveal any significant correlations (right FFA: r = 0.07, t(13) = 0.252, p > 0.8; left FFA: r = 0.084, t(10) = 0.267, p > 0.75; see Supplementary Material for further details).

We further examined the structural correlates of car expertise in an independent analysis by applying a non-parametric bootstrapping technique (FSL TFCE, see Methods) to our data. This additional whole brain corrected analysis (p < 0.05 corrected) revealed only one significant region (p = 0.006) again outside the visual cortex, which overlapped the region found in the right inferior precentral sulcus and bordering the upper insula in the VBM analysis.

To explore the correlation between neuroanatomical structure and visual recognition performance that was not dependent on car expertise, we applied VBM analysis to correlate the local gray matter volume with performance on the control category of airplanes, with which, based on our selection criteria, none of our participants was expert. This analysis revealed that performance on the airplane task was correlated with gray matter volume of regions in or bordering the visual cortex including the right parietal cortex, and the right fusiform (see Fig. 2b and Table 1). The FSL bootstrapping technique did not reveal any brain region whose gray matter volume was correlated with performance on the control category of airplanes.

An additional VBM analysis to examine possible neuroanatomical correlates of the joint behavioral measures (car and airplane performance) replicated the results described above for car and airplane performance separately, with no additional regions being implicated (cf. Table 1 and Supp. Table 1).

## Discussion

We investigated how acquired real-world expertise with a specific object category such as cars, affects cortical neural structure. To address this question we examined whether variability in the gray matter volume of different brain regions is reliably associated with the level of car recognition expertise. Specifically, we examined whether correlations between visual car expertise and gray matter structure occurred only in regions of the ventral occipito-temporal cortex that are involved in object shape analysis or also in structures outside of ventral occipito-temporal cortex. Our present findings supported the latter possibility better. In fact, our results showed that expertise with visual recognition of car models is associated with changes in local prefrontal cortex structure as well as bordering the upper insula, but not with regions in the ventral occipito-temporal cortex. Thus, although the kind of expertise demonstrated by our participants was in recognizing and visually matching the models of cars, changes in neural structure associated with their expertise were found outside the ventral occipito-temporal cortex. This finding suggests that expertise for real-world objects accumulated through learning does not necessarily entail structural changes in regions associated with visual processing or visual object representations per se, but rather in more anterior regions particularly in the prefrontal cortex, which is associated, among other functions, with cognitive control of semantic processing (for a review see (Binder et al., 2009)). This pattern suggests top-down involvement of higher-level mechanisms in expert visual processing, probably reflecting the increased semantic knowledge about objects in the domain of expertise.

We found no statistically significant evidence for an association between the gray matter volume of ventral visual cortex and visual car expertise. It could be the case that the absence of such significant correlation stemmed from a lack of statistical power. However, several reasons suggest that it did not. First, we did not find any correlation between visual expertise and the neural structure of the ventral visual cortex even at a much more liberal (uncorrected) statistical threshold. Second, there was a statistically significant association between visual car expertise and the neural structure of prefrontal regions at the more stringent statistical threshold. Finally, our results were validated by two different independent methods of analysis, the voxel-based morphometry (VBM) method (Ashburner and Friston, 2000), and the threshold-free cluster enhancement (TFCE) method in FSL (Smith and Nichols, 2009). Importantly, both analyses independently revealed correlations between gray matter structure and car expertise performance in prefrontal cortex, specifically within a region in precentral sulcus. The inclusion of the non‐parametric permutation-based TFCE analysis was particularly important, as it minimized the possibility that the clusters identified in the current study resulted from non-stationarity in the data (Salimi-Khorshidi et al., 2011).

Car expertise has been shown in several fMRI studies to selectively engage the fusiform gyrus (Gauthier et al., 1999, 2000a, 2000b, 2005; Wong et al., 2009; Xu, 2005) and (McGugin, Gatenby, Gore, & Gauthier, unpublished). On the basis of such findings it has been assumed that expertise reflects a change in visual processing strategies (for example, from part-based to global- prioritized strategies, cf., (Gauthier and Tarr, 2002; Gauthier et al., 2003; Richler et al., 2011; Wong and Gauthier, 2010b). While the pattern of fMRI activation seen in these other studies might support this view (but see Grill-Spector et al., 2004; Harel et al., 2010 for alternative patterns), here we wanted to examine whether expertise with object recognition might exert a similar effect on gray matter structure of ventro-occipital temporal cortex.

While we found no statistically significant evidence for expertise-associated changes in gray matter volume of ventral visual cortex, our results do suggest that long-term acquired (visual) expertise for objects can induce structural changes in the prefrontal cortex. This finding is consistent with a previous study, which shows that disruption to white matter tracts connecting the ventral visual cortex to prefrontal regions (including the ones found in the current study) can cause visual recognition deficits (Thomas et al., 2009). Neuropsychological evidence also supports a role for prefrontal regions in visual recognition. Firstly, brain damage to prefrontal regions can cause deficits in visual recognition (Damasio et al., 2004). Secondly, patients with extensive damage to ventral occipito-temporal cortex can nevertheless retain their pre-morbid expertise for cars (Sergent and Signoret, 1992) or acquire novel visual expertise (McNeil and Warrington, 1993)^2^. Finally, the anterior left IFG (see Table 1) is also involved in controlling retrieval of individuated information in particular knowledge domains (Badre and Wagner, 2007). The present results might suggest that the PFC is also associated with mediating the effects of expertise on visual object recognition.

What does finding an association between the neuroanatomy of prefrontal regions and long-term visual expertise teach us about expertise in general? We hypothesize that the prefrontal regions delineated in our study play a role in accessing and processing individuated visual knowledge leading to familiarity. In other words, when someone becomes a perceptual expert in a specific visual category, this is accompanied by the acquisition of vast personal visual knowledge related to that category, including knowledge related to the shape characteristics of individual objects from the domain of expertise (see also Barton et al., 2009). If the knowledge about visual items leads to the formation of enriched and distinctive visual representations, those representations might be stored in the perceptual semantic system that is accessed and processed by prefrontal regions (Binder and Desai, 2011). Note, however, that individuals with different visual specializations would have different visual categories controlled or represented in these regions. Thus, our hypothesis suggests that the regions identified in our present study are not necessarily specific for car expertise, but would exhibit similar relationships with other types of expertise knowledge developed for visual objects in other categories.

Our findings might reflect the type of expertise tested in this study. It is conceivable that the task we selected required conceptual knowledge about cars as well as car-related expert perception. Thus, the performance that we correlated with brain structure in this study might have included pre-existing semantic knowledge as well as expert perceptual abilities. However, in real‐world expertise it is often impossible to separate the semantic and perceptual contributions (Barton et al., 2009). Thus, although voluntary acquired expertise for real-world objects probably involves changes in semantic knowledge and conceptual representations (Johnson and Mervis, 1997; Rosch and Mervis, 1976), it probably cannot be purely conceptual and, hence, the possibility that the selection of experts in the present study has been biased to specifically exclude perceptual expertise is not very likely.

Unlike cars, there was no significant correlation between performance in the airplane make-matching task and gray matter volume in prefrontal regions. Since our participants were not airplane experts, they probably did not have conceptual representations of different airplane brands. Hence, their performance might have been based on direct visual comparison. If that is the case, then our results, that performance levels in the airplanes task were correlated with changes in the neural structure of the visual cortex is not surprising.

In summary, the present findings demonstrate that, as for other domains of sensorimotor expertise (Gaser and Schlaug, 2003; Scholz et al., 2009), expertise in visual recognition can be associated with variation in the local structure of highly focal regions in the frontal lobe. Thus, it appears that structural changes in the human brain are a common motif of expertise, regardless of whether this involves motor or visual performance. It remains an open question whether this structural variability across participants is also associated with variability in activation of these areas either within or across participants while expertise is exerted. Indeed, it may be possible to reconcile earlier findings that BOLD signals in the ventral visual cortex vary with expertise ((Gauthier et al.,1999, 2000b, 2005; Wong et al., 2009; Xu, 2005) and (McGugin, Gatenby, Gore, & Gauthier, unpublished)) and our finding in the current study that areas of prefrontal cortex show structural co-variation with car expertise, if structural and functional correlates of expertise are regionally dissociated. Conjoint study of structure and function examining co-variations with expertise will therefore be an important area to address in future research.

## Conflict of Interest

The authors declare no conflicts of interest.

## Figures and Tables

**Table 1:** Details of brain regions where gray matter density significantly correlated with visual expertise for cars (top panel, corresponding to Fig. 1a), or with a control non-expertise category (planes, bottom panel, see Fig. 1b). No regions were correlated with the interaction of these two factors (see Methods and Supplementary Table 1). MNI coordinates in mm. Cluster size in mm^3^.

	Anatomy	MNI coordinates			Cluster size	F_(1,16)_	Z	P (corrected)
X	Y	Z
Visual expertise (cars)	Right inferior precentral sulcus (R-iPC)	53	2	3	538	92.44	5.34	0.007
Anterior left inferior frontal gyrus (L-aIFG)	− 44	36	0	162	56.03	4.70	0.005
Right superior frontal gyrus (R-SFG)	14	57	13	42	47.65	4.49	0.012
Right middle frontal gyrus	32	21	39	14	36.63	4.15	0.026
Control category (planes)	Right parietal cortex (R-IPS)	18	− 63	52	68	40.17	4.27	0.025
Right fusiform (R-FG)	42	− 42	− 21	202	40.60	4.28	0.022

## Footnotes

2 Busigny, T., M. Graf, E. Mayer and B. Rossion, 2010. Acquired prosopagnosia as a face-specific disorder: ruling out the general visual similarity account. Neuropsychologia. 487, 2051–2067. discusses various cases of ventral visual cortex damage affecting premorbid object recognition expertise
