# Supplementary material for: Neuroanatomical correlates of visual car expertise
Source: Neuroimage. 2012 Aug 1;62-334(1):147–53. doi: 10.1016/j.neuroimage.2012.05.017 (PMC3387385; doi:10.1016/j.neuroimage.2012.05.017)
Supplement: Supplementary file 1 — Supplementary materials [file mmc1.doc]

**Supplementary Information**

**Neuroanatomical correlates of visual car expertise**

Authors:

Sharon Gilaie-Dotan, Assaf Harel, Shlomo Bentin, Ryota Kanai, Geraint Rees

Contents:

Supplementary Methods

Supplementary Results

Supplementary Table 1

Supplementary Figure 1

**Supplementary Methods**

*Participants*

The twelve car experts and nine car novices whose data are reported in this study have all participated in a previous study . That study included 13 experts and 15 novices. Due to technical reasons 7 participants that participated in that study (1 expert and 6 novices) were not included in the current study: 3 participants (1 expert and 2 novices) were not included since their structural scans were from a different scanner (1.5T), and 4 participants (novices) were not included since their behavioural data were not successfully retrieved. All the structural scans that were analyzed in the current study were collected in the same scanning sessions of the Harel et al. (2010) study.

*Functional localization of the FFA*

Localization of the face-selective Fusiform Face Area (FFA) region of interest (ROI) was based on the functional scans reported in detail in the [Harel et al. (2010](#_ENREF_23)) study. Face-selective region in the posterior aspect of the right fusiform gyrus (FFA-R) was localized in 15 out of the 21 participants of this study, and in 12 participants in the left fusiform gyrus (FFA-L). The independent functional localizer experiment was block designed and included 4 conditions: faces, houses, objects, and textures. Face-selective voxels were defined by preference to faces over houses (for further details see [Harel et al., 2010](#_ENREF_23)). The FFA ROIs from the previous study, which were in Talairach normalized space, were converted to MNI normalized space to be used in the current study.

*Correlating neuroanatomical structure of FFA with car expertise*

For each participant that had a functionally-defined FFA (right or left), we extracted the gray matter (GM) density from all voxels in that ROI. The average GM density over all the voxels in that ROI was taken as the GM density value to be correlated with behavioral performance.

**Supplementary Results**

To increase our sensitivity to detect an effect of expertise in the structure of the face-sensitive region in the fusiform gyrus (FFA), we examined whether the gray matter structure of an independently functionally defined FFA was associated with car expertise. We sampled the gray matter density of the right and of the left FFA (FFA-R and FFA-L respectively) and correlated them with car expertise (see figure below, top panels). No significant correlations were found (FFA-R: r = 0.07, t(13) = 0.252, p > 0.8; FFA-L: r = 0.084, t(10) = 0.267, p > 0.75). We performed the same analysis with performance on the control category of planes and found significant correlations for FFA-L (r = 0.597, t(10) = 2.352, p =0.0405), but not for FFA-R (r = -0.378, t(13) = -1.473, p = 0.165). These appear in the lower panels of the figure below. No significant difference was found between the correlation of car expertise and FFA’s gray matter density, and the correlation of plane performance and FFA’s gray matter density (FFA-R: Fisher’s z: 1.15, p > 0.25; FFA-L: Fisher’s z: -1.28, p > 0.2 ).

**Supplementary Table 1**

In order to examine whether there are brain regions whose neural structure correlates with the interaction between car expertise level and the performance on the control category of planes, in this analysis both measures were inserted into the F contrast. These results are consistent with the ones presented in Table 1, no additional regions were found. MNI coordinates in mm. Cluster size in mm^3^.

|  | Anatomy | MNI coordinates | | | Cluster size | F_(2,16)_ | Z | P (corrected) |
| --- | --- | --- | --- | --- | --- | --- | --- | --- |
|  |  | X | Y | Z |  |  |  |  |
| Visual expertise (cars) | Right inferior pre-central sulcus (R-iPC) | 53 | 2 | 3 | 343 | 46.55 | 5.06 | 0.012 |
|  | Anterior left inferior frontal gyrus  (L-aIFG) | -44 | 36 | 0 | 67 | 35.23 | 4.69 | 0.006 |
|  | Right superior frontal gyrus (R-SFG) | 14 | 57 | 13 | 18 | 24.11 | 4.18 | 0.032 |
|  | Right middle frontal gyrus | 32 | 21 | 39 | 8 | 22.41 | 4.08 | 0.026 |
| Control category (airplanes) | Right parietal cortex (R-IPS) | 18 | -64 | 53 | 69 | 25.19 | 4.24 | 0.015 |
|  | Right fusiform (R-FG) | 41 42 | -36  -42 | -14  -21 | 169 | 22.31 20.32 | 4.07 3.94 | 0.022 |

**Supplementary Figure 1**

The results in this figure are identical to those presented in Figure 2 in the main text. For display purposes only, novices are indicated in lighter colors and car experts in darker colors. Note that the structural VBM analysis treated all participants as a continuum and *did not* distinguish between novices and experts groups. Several of the experts performed an initial version of the car discrimination task, which used less familiar car models and therefore scored slightly lower.
